# Effects of Ischemic Compression on Trigger Points in the First Dorsal Interosseous Muscle in Patients with Thumb Carpometacarpal Osteoarthritis

**DOI:** 10.3390/ijerph18062961

**Published:** 2021-03-14

**Authors:** María Pilar López-Royo, Paolo Pedersini, Raquel Cantero-Téllez, Kristin Valdes, Víctor Doménech-García, Pablo Herrero, Jorge Hugo Villafañe

**Affiliations:** 1 iPhysio Research Group, Universidad San Jorge, Autov. A23 km 299, Villanueva de Gállego, CP 50830 Zaragoza, Spain; mplopez@usj.es (M.P.L.-R.); vdomenech@usj.es (V.D.-G.); 2 IRCCS Fondazione Don Carlo Gnocchi, 20148 Milan, Italy; ppedersini@dongnocchi.it (P.P.); jhvillafane@dongnocchi.it (J.H.V.); 3 Tecan Hand Center, University of Málaga, CP 29009 Málaga, Spain; cantero@uma.es; 4 Ruskin Campus, Gannon University, Ruskin, FL 33573, USA; Valdes001@gannon.edu; 5 Department of Physiatry and Nursing, Faculty of Health Sciences, University of Zaragoza, C/Domingo Miral s/n, CP 50009 Zaragoza, Spain

**Keywords:** myofascial pain, myofascial trigger point, osteoarthritis, pain management, pressure pain threshold

## Abstract

Background: Thumb carpometacarpal osteoarthritis (CMC OA) is a common disorder that interferes with the ability to perform the activities of daily life. The purpose of this study was to investigate the immediate effects of ischemic compression on myofascial trigger points (MTrPs) in the first dorsal interosseous (FDI) muscle in patients with the diagnosis of thumb CMC OA. Methods: In a quasi-experimental clinical trial, thirty-one patients, 87% female (age: 82 ± 9.4 years), with thumb CMC OA, were consecutively assigned to either an experimental treatment that included the ischemic compression of the FDI MTrP or a sham treatment of the FDI MTrP for one session. The main outcome considered in the study was the pressure pain threshold (PPT). Measurements were taken pre- and post-treatment and at a 1-week follow-up period. Results: The PPT over the right (affected) FDI muscle showed statistically significant differences between groups at 1-week follow up (F = 3.518; *p* = 0.04) in favor of the experimental group. Conclusions: The ischemic compression of FDI-MTrPs is an appropriate part of a multimodal treatment to decrease local pain sensitivity in patients with CMC OA.

## 1. Introduction

Thumb carpometacarpal osteoarthritis (CMC OA) is a common disorder that interferes with the ability to perform activities of daily living [1]. It is the most often reported painful joint compared to all other hand joints [2], and it is present in 40% of women and up to 25% of men older than 75 years of age. Its diagnosis is based on symptoms such as the location of pain, tenderness and instability, as well as radiographic evaluation [3]. Potential intrinsic, posttraumatic and abnormal biomechanical forces have been reported to increase the probability of the development of CMC OA [4]. The patient with CMC OA reports pain and weakness that can be a consequence of CMC ligamentous laxity and is associated with joint hypermobility.

The first dorsal interosseous muscle (FDI) abducts the index finger. Eyler at al. [5] and Masquelet et al. [6] reported that the FDI muscle also adducts the first CMC joint. Recently, it has been shown that the contraction of the dynamic stabilizers (mainly FDI) reduces the subluxation of the CMC and thus the load on the joint, so we can hypothesize that conservative treatment focused on the FDI muscle could be key in approaching this pathology [7,8].

Myofascial trigger points (MTrP) are an extremely prevalent cause of persistent pain disorders in all parts of the body [9]. MTrPs are highly irritable areas in tight bands of skeletal muscle that are painful when compressed and can elicit referred pain [10]. Although no specific studies have examined the effectiveness of ischemic compression on MTrPs in patients with thumb CMC OA, we hypothesize that this treatment may have a potential effect on both local and widespread sensitivity. Therefore, the purpose of this study was to investigate the immediate effects of ischemic compression of MTrPs in the FDI muscle in patients with the diagnosis of thumb CMC OA on both local and widespread sensitivity.

## 2. Methods

### 2.1. Study Design

We conducted a double blind (evaluator and statistician) prospective quasi-experimental clinical trial. The STROBE published guidelines were used to guide the study design of the trial [11].

### 2.2. Ethical Consideration

Prior to the initiation of treatment, the participants provided informed consent, and all study procedures were conducted according to the Declaration of Helsinki. This research protocol was approved by the Local Ethical Committee of “IRCCS Fondazione Don Carlo Gnocchi”, Italy, on 24 February 2016.

### 2.3. Participants

Participants with a medical diagnosis of right thumb CMC OA (grades 3 to 4 using the Kellgren and Lawrence grades), a positive clinical grind test for CMC OA, and positive radiographic findings [12] were recruited at Fondazione Don Carlo Gnocchi at Rovato (Italy) from July to November 2017. The exclusion criteria were having a past medical history of injuries or previous lesions to the cervical spine, carpal tunnel syndrome, fibromyalgia syndrome, hand surgery, De Quervain’s tenosynovitis or with degenerative or non-degenerative neurological conditions in which pain perception was altered. None of the individuals in this study had received prior conservative or surgical interventions for CMC OA. A priori power calculation was performed to determine sample size. The calculation was based on the results of other studies of thumb CMC OA patients, to detect a difference in reliability of 0.98, at 80% power, and a 5% level of significance. The a priori power calculation determined that 15 individuals were needed in each group (experimental and control).

### 2.4. Assessments and Procedure

A physical therapist [PP] trained specifically in the protocol who was blinded to group assignment and to the purpose of the study assessed the patients and carried out the outcome measurements at baseline (Pre), immediately post-intervention (Post) and at 1-week follow up (FU). Baseline assessments included gender, age, dominant hand, pressure pain threshold (PPT) [13], the brief version of the Disabilities of the Arm, Shoulder and Hand (Quick-DASH), the Visual Analogue Scale (VAS) and the key pinch strength [14].

The PPT was assessed as a baseline variable but was also considered the main outcome for the study. PPT is a quantitative sensory test of tissue sensitivity and has been described as the least amount of pressure applied that produces pain [13]. PPT was measured via a pressure algometer (Force dial FDK 20, Wagner) [13] and was evaluated bilaterally on the hand, first at the CMC joint, followed by the FDI. The patients were asked to tell the examiner the exact moment when the pressure started to change into a pain sensation, in order to determine at which moment to stop applying pressure. Two measurements were attained from each point, and the mean was calculated.

Regarding the rest of the baseline assessments, the Quick-DASH questionnaire was used to assess the patients’ self-reported upper extremity function [14]. The patients’ key pinch strength was assessed with a mechanical pinch gauge (Baseline, NY, USA) with the patient sitting with their shoulder adducted, forearm in neutral rotation and the elbow flexed to 90° [15].

After completion of the outcome questionnaires and baseline data collection, participants were consecutively assigned to either the experimental or sham group by a different physical therapist (MPL) who was blinded to the baseline assessments. The physical therapist (MPL) diagnosed the MTrPs in the FDI muscle and performed the interventions. To diagnose MTrPs, the patient laid on a mat in a supine position with the forearm held in a neutral position, while the physical therapist (MPL) palpated the muscles perpendicular to the fibers’ direction looking for taut bands and progressed longitudinally along the taut band to determine if there was a painful area or nodule in the taut band, and if the painful area corresponded with a nodule [16]. When a MTrP was diagnosed, the physical therapist (MPL) performed a compression test, which consisted of applying a sustained manual pressure for twenty seconds and then asked the patient if they had any referred pain, to classify the MTrP as either active or latent [16]. If the compression test reproduced referred pain that was familiar for the patient, the MTrPs were considered active. During all the palpations when a hypersensitive nodule was found in the FDI, the therapist asked the following questions with a yes or no response.

- Is there a taut band?
- Is there a hypersensitive point?
- Is there a nodule coinciding with the hypersensitive point?
- Does compression provoke referred pain familiar to the patient?

**Figure 1:**
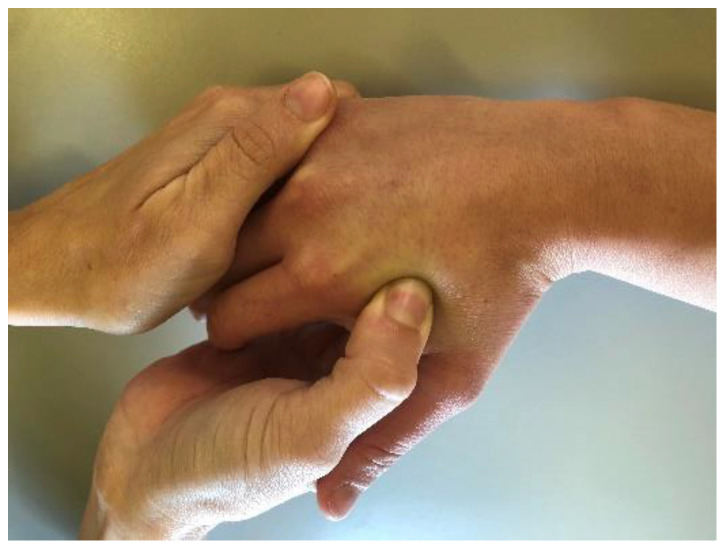
Ischemic compression over the MTrP of the FDI.

After diagnosing MtrPs, the experimental group received a treatment that consisted of an ischemic compression over the MTrP of the FDI for 1 min, until the subject experienced pain. The sham/control group received a treatment which consisted of a digital touch applied (without pressure) to the MTrP of the FDI for 1 min [Figure 1]. After the treatment, patients were evaluated again by the blinded physical therapist (PP), who measured the PPT on the bilateral CMC joints and bilateral FDI MTrPs. After the experimental or sham treatment, participants in both groups received usual care consisting of a passive range of motion of the hand and exercises once a day for one week.

### 2.5. Statistical Analysis

Data were analyzed with SPSS for Windows (V.22, IBM, Armonk, NY, USA). The results are expressed as the means and standard deviations (SDs). The Kolmogorov–Smirnov test was used to determine if there was a normal distribution of the data. A two-way, mixed-model ANOVA (PPTs (MTrP in FDI muscle, first at the CMC joint, side (dominant, non-dominant), time point (pre, post-treatment and FU)) was applied to determine the effect of ischemic compression applied to the MTrP of the FDI muscle or the CMC joint OA. Where appropriate, univariate contrast and Bonferroni-adjusted post-hoc analyses were performed. The analysis of variance (ANOVA) was used to determine the difference over time (pre, post-treatment and FU) and to compare the outcome mean scores of the within-subjects factor and group (experimental or sham) as the between-subjects factor. The statistical analysis was conducted at a 95% confidence level, and *p* < 0.05 was considered statistically significant. Mauchly’s sphericity test was carried out to validate a repeated measures analysis of variance.

## 3. Results

### 3.1. Demographic and Clinical Data of Participants

Thirty-one (n = 31) patients with thumb CMC OA (mean ± SD age: 82 ± 9.5 years; 87% female) agreed to participate in the study and met all eligibility criteria. Sixteen patients were assigned to the experimental group and 15 patients to the sham group. None of the subjects modified their regular pharmacologic treatment during the study. No participants dropped out during the study or reported adverse effects after the treatment. The baseline characteristics were similar between groups (Table 1).

### 3.2. Pressure Pain Sensitivity

The ICCs for the intraexaminer reliability of PPT measurements ranged from 0.938 to 0.990 for the affected side and from 0.913 to 0.977 for the unaffected side. The SEMs ranged from 0.07 to 0.19 kg/cm^2^ for both sides.

For PPTs measured over the first CMC joint, the 2 × 2 × 3 ANOVA revealed no significant group × time × side (F = 0.559; *p* = 0.575), group × time (F = 0.694; *p* = 0.504), side × time (F = 1.718; *p* = 0.189) or group × side (F = 1.618; *p* = 0.215) interactions (Table 2). There was also no significant effect of time (F = 0.883; *p* = 0.420) or side (F = 0.023; *p* = 0.880).

For PPTs measured over the FDI-MTrP, the 2 × 2 × 3 ANOVA revealed no statistically significant interaction for group × time × side (F = 1.632; *p* = 0.216), side × time (F = 1.618; *p* = 0.218) or group × side (F = 0.863; *p* = 0.361). However, there was a statistically significant group × time interaction (F = 3.518; *p* = 0.04) between the mean scores of the patients that received the experimental protocol exhibiting greater PPT over the right FDI-MTrP (*p* < 0.024) as compared to those that received the sham treatment at the 1-week FU.

## 4. Discussion

The present study showed for the first time that ischemic compression of the FDI-MTrP led to a local reduction in the pressure pain sensitivity. Different studies have shown that the compression of MTrPs increases the PPTs. For example, the study conducted by Ziaeifar et al. showed a PPT increase after upper trapezius MTrP compression [17]. In line with this, the study conducted by Sohns et al. [18] showed that both manual MTrP compression therapy and sham manual therapy led to an increase in the shoulder PPTs, although the increase was higher in the experimental group. These results contrast with our research where no difference between groups on the affected side was found for the FDI PPTs immediately after the compression, but rather at the 1-week FU.

However, our results are in line with those of De Meulemeester et al., who did not find changes in the PPTs immediately after the manual compression of shoulder MTrPs but found significant changes after 4 weeks of treatment [19]. Little is known about the working mechanisms underlying the effects of manual compression. It has been hypothesized that compression effects may depend on reactive hyperemia, counter-irritant effects or a spinal reflex mechanism resulting in a release of muscle spasm [20]. However, another possible reason for the differences between studies could be the degree of sensitization, which may depend on the baseline levels of pressure pain sensitivity. In our study, we were not able to compare our results with other studies as there are no previous studies measuring the PPTs in the FDI muscle. Nevertheless, we hypothesize that the low PPTs existing in patients with CMC OA could explain the lack of effects immediately after manual compression.

There is some research regarding the evidence that central sensitization plays a role in CMC OA chronic and recurring pain [21]. In our study, the beneficial effects of localized pressure to reduce hypersensitivity at the FDI region are limited. A positive response was obtained after 1 week and only for the affected side, which points to the possibility that manual compression techniques of MTrPs may play a role in the local, but not in the widespread, sensitivity in patients with thumb CMC OA.

## 5. Conclusions

The ischemic compression of FDI-MTrPs is an appropriate part of a multimodal treatment to decrease local pain sensitivity in patients with CMC OA. Further studies including a control group and longer treatments are needed to better understand the effects of the ischemic compression of MTrPs in patients with CMC OA.

## Figures and Tables

**Table 1:** Baseline demographics for both groups *.

	Exp Group (n = 16)	Con Group (n = 15)
Age (n, mean ± SD)	82.6 ± 9.5	81.40 ± 8.3
Gender, *female* (n, %)	15, 93%	12, 80%
Dominant hand, *right* (n, %)	16, 100%	15, 100%
Quick-DASH	21.7 ± 11.7	21.8 ± 13.4
Key pinch, *right (affected)*	3.1 ± 1.4	3.4 ± 1.8
Key pinch, *left (non-affected)*	3.0 ± 1.3	3.3 ± 1.9
VAS-key pinch, *right (affected)*	1.1 ± 2.3	1.1 ± 2.6
VAS-key pinch, *left (non-affected)*	0.9 ± 1.8	0.6 ± 2.0
VAS.24, *right (affected)*	0.7 ± 1.7	0.7 ± 1.6
VAS.24, *left (non affected)*	0.7 ± 1.6	0.4 ± 1.3
PPT Findings (n, mean ± SD)		
FDI Muscle, *right (affected)*	1.8 ± 0.7	1.8 ± 0.6
FDI Muscle, *left (non affected)*	2.0 ± 0.7	1.8 ± 0.6

* Data are expressed as means ± standard deviations (SD); VAS: visual analogue scale; PPT: pressure pain threshold.

**Table 2:** Mean (SD) for pressure pain thresholds at all study visits for each group; mean (SD) difference within groups.

Outcome	Pre				Difference within Groups							
PPT (kg/cm^2^)					Post Minus Pre				FU Minus Pre			
	Exp		Con		Exp		Con		Exp		Con	
	(n = 16)		(n = 15)		(n = 16)		(n = 15)		(n = 16)		(n = 15)	
	Right	Left	Right	Left	Right	Left	Right	Left	Right	Left	Right	Left
First CMC joint	2.0	2.0	2.2	2.1	0.05	−0.2	0.2	0.1	0.1	0.02	0.2	0.08
	(0.7)	(0.4)	(0.6)	(0.6)	(0.2)	(0.1)	(0.2)	(0.1)	(0.1)	(0.1)	(0.1)	(0.1)
MTrP-FDI	1.6	1.9	1.8	1.9	0.2	0.0	0.1	0.0	0.3 *	0.0	0.1	0.1
	(0.4)	(0.3)	(0.7)	(0.5)	(0.1)	(0.1)	(0.1)	(0.1)	(0.6)	(0.7)	(0.1)	(0.1)

Exp = experimental group; Con = control group; CMC = carpo-metacarpal joint; PPT = pressure-pain threshold; FU (follow up). * Right hand (affected); left hand (non-affected).

## Data Availability

The data presented in this study are available on request from the last author.
